# The Mediating Role of Biological Age Advance in the Association Between Periodontitis and Mortality: **Biological Aging Links Periodontitis to Mortality**

**DOI:** 10.1002/cre2.70305

**Published:** 2026-02-09

**Authors:** Dawei Zhang, Shijie Zhu, George Pelekos, Lijian Jin, Patrick Rijkschroeff

**Affiliations:** ^1^ Division of Periodontology and Implant Dentistry, Faculty of Dentistry The University of Hong Kong Hong Kong China; ^2^ School of Public Health, LKS Faculty of Medicine The University of Hong Kong Hong Kong China; ^3^ Quality Management and Accreditation Department The University of Hong Kong‐Shenzhen Hospital Shenzhen China

**Keywords:** biological age, mortality, periodontitis

## Abstract

**Objectives:**

This study aims to test a conceptual mediation model wherein periodontitis is associated with mortality through direct pathways and indirectly via accelerated biological aging.

**Material and Methods:**

We analyzed six cycles of National Health and Nutrition Examination Survey data with mortality follow‐up of 250 months. Weighted descriptive statistics were used to compare group characteristics, Kaplan–Meier analysis to evaluate periodontitis‐mortality associations and generalized linear models to examine the links between periodontitis and biological aging. Cox proportional hazards models integrated with restricted cubic splines were utilized to explore the association between biological age and mortality, and mediation analyses quantified the mediation of biological aging. Additionally, age, gender, and smoking status subgroup analyses were conducted.

**Results:**

Moderate/severe periodontitis was associated with a significantly elevated all‑cause mortality risk (18.31% vs. 10.88% in no/mild periodontitis) and greater biological age advancement (PhenoAge: 1.22 years; KDM: 0.68 years). Biological age acceleration exhibited a non‐linear association with mortality, with hazard ratios rising sharply beyond a threshold (PhenoAge: 16.4 years; KDM: 31.8 years). Mediation analysis showed that biological age partially mediated the periodontitis–mortality association, with indirect effect hazard ratios of 1.085 (95% CI: 1.067–1.106) for PhenoAge advance and 1.027 (95% CI: 1.016–1.040) for KDM advance in all‑cause mortality, though the proportion mediated was modest and varied across subgroups.

**Conclusions:**

Our findings support the hypothesis that biological aging (assessed by PhenoAge and KDM advances) plays a significant, though partial, mediating role in the link between periodontitis and elevated mortality.

## 1 Introduction

Periodontitis is characterized by chronic inflammation of the tooth‐supporting tissues. It affects more than 60% of people globally (Trindade et al. 2023), with up to 1.1 billion of people experiencing severe forms (Chen et al. 2021). Beyond oral health impairment, mounting evidence highlights periodontitis as a latent risk factor for systemic diseases (Villoria et al. 2024; Zhao et al. 2019). Furthermore, epidemiological studies consistently link periodontitis to higher risks of all‐cause and cause‐specific mortality. These include mortality related to cardiovascular disease (CVD), cancer, and respiratory disease (Larvin et al. 2024; Romandini et al. 2020). Notably, this adverse relationship has also been validated in some high‐risk populations, such as individuals with CVD (Chen et al. 2024), hyperlipidemia (Xu et al. 2024), hypertension (Li, Yao et al. 2024), chronic kidney disease (Li et al. 2023), and depression (Zhang et al. 2024).

The mechanisms linking periodontitis to mortality remain unclear, though systemic inflammation has been proposed as one pathway. However, a 17‐year prospective cohort study found that systemic inflammation (measured by C‐reactive protein) mediated only about 10% of this association (Winning et al. 2021). Beyond systemic inflammation, senescence has emerged as another plausible mediator (Liu et al. 2022). Accumulating evidence suggests that periodontitis is linked to accelerated biological aging (Li, Wen et al. 2024; Huang et al. 2024). Unlike chronological age (CA), biological age reflects cumulative physiological deterioration, which is closely associated with disease onset and mortality risk (Rutledge et al. 2022).

Among metrics of biological age, phenotypic age (PhenoAge) and Klemera–Doubal method (KDM) have emerged as robust predictors of adverse health outcomes (Levine et al. 2018; Klemera and Doubal 2006). Both methods utilize regression models and incorporate CA alongside multiple clinical biomarkers, yet they differ in their core principles (Levine et al. 2018; Klemera and Doubal 2006). PhenoAge employs an elastic‐net Gompertz regression framework to integrate biomarkers into a mortality risk score. This score is then converted into a phenotypic age by aligning it with the mortality risk of a reference population, ultimately reflecting the CA at which an individual's mortality risk matches the population average (Levine et al. 2018). In contrast, KDM estimates biological age by integrating regression parameters from linear models of individual biomarkers against CA. It uses weighted combinations to minimize deviation from a reference regression line (Klemera and Doubal 2006).

**Figure 1:**
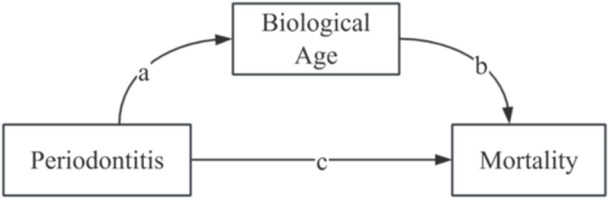
Conceptual model of the mediation effect. This diagram illustrates the hypothesized pathways by which periodontitis is associated with mortality. Path a represents the association of periodontitis with biological age; path b represents the association of biological age on mortality (the potential mediating pathway); and path c represents the direct association of periodontitis on mortality.

Despite the reported modification role of periodontitis in linkage between biological age and mortality in a previous study (Liu et al. 2023), here we focus on the mediating role of biological age in the periodontitis‐mortality pathway. Leveraging the nationally representative National Health and Nutrition Examination Survey (NHANES) data with long‐term mortality follow‐up, this study tests a conceptual mediation model (Figure 1), wherein periodontitis influences mortality risk indirectly through accelerated biological aging (assessed by both PhenoAge and KDM). Specifically, we aim to determine whether periodontitis associates with increased all‐cause and cause‐specific mortality via the mediation of biological aging.

In spite of prior investigations into the periodontitis–biological aging relationship (Li, Wen et al. 2024; Huang et al. 2024; Nguyen et al. 2022, 2025), the present study employs a distinct approach by integrating dual biological age metrics within a nationally representative NHANES sample from 1999 to 2014, incorporating extended 250‐month mortality follow‐up, performing mediation analyses for all‐cause and cause‐specific mortality, and conducting subgroup analyses by age, gender, and smoking status to explore underlying pathways.

## 2 Materials and Methods

### 2.1 Study Population

This retrospective cohort study utilized data from six continuous cycles (1999–2000, 2001–2002, 2003–2004, 2009–2010, 2011–2012, 2013–2014) of the NHANES, conducted by the National Center for Health Statistics (NCHS). NHANES employes a stratified, multistage probabilistic sampling design to generate nationally representative data through standardized interviews and physical examinations. All study protocols were approved by the NCHS Ethics Review Board. This study was reported in accordance with the Strengthening the Reporting of Observational Studies in Epidemiology (STROBE) Statement (Table S1). Participants with missing data on key variables (e.g., periodontal examination, biomarkers for biological age calculation, mortality status, or covariates) were excluded.

### 2.2 Periodontal Examination and Definitions of Periodontitis

Licensed dentists performed comprehensive full‐mouth periodontal assessments for participants aged ≥ 30 years within dedicated mobile examination center facilities. Periodontal examination protocols varied across NHANES cycles. During the 1999–2004 cycles, a partial‐mouth periodontal examination protocol was employed, assessing a random half‐mouth (one upper and one lower quadrant) with probing depth (PD) and clinical attachment loss (CAL) measured at three sites per tooth: distal‐facial interproximal, mid‐facial, and mesial‐facial interproximal. In contrast, the 2009–2014 cycles utilized a full‐mouth periodontal examination (FMPE) protocol, recording standardized measurements of PD and gingival recession (to derive CAL) across six sites per tooth (mesio‐facial, mid‐facial, disto‐facial, mesio‐lingual, mid‐lingual, disto‐lingual), excluding third molars. To minimize measurement bias, a comprehensive quality assurance protocol was implemented including standardized examiner training, automated real‐time data validation, and annual blinded audits with duplicate examinations by reference clinicians. All collected PD and CAL metrics were subsequently classified according to the CDC‐AAP periodontitis severity staging system to determine disease status (Eke et al. 2012).

### 2.3 Assessment of Mortality

Mortality data with follow‐up time ending on December 31, 2019 were obtained from the public‐use Linked Mortality Files compiled by the NCHS, which linked data from NHANES to death certificate records in the National Death Index (Continuous NHANES Public‐use Linked Mortality Files 2019). Underlying cause of death was coded according to the International Classification of Diseases, 10th Revision (ICD‐10). Cause‐specific mortality was classified as: cancer mortality (ICD‐10 codes C00–C97) and CVD mortality (ICD‐10 codes I00–I09, I11, I13, I20–I51, I60, and I69).

### 2.4 Biological Age Calculation

Biological age was quantified using both PhenoAge and KDM algorithms (kdm_calc and phenoage_calc) implemented in the BioAge R package with a standardized biomarker panel comprising albumin (g/L), alkaline phosphatase (U/L), total cholesterol (mg/dL), creatinine (μmol/L), glycated hemoglobin (HbA1c) (%), systolic blood pressure (mmHg), blood urea nitrogen (mg/dL), urine microalbumin (mg/L), lymphocyte percentage (%), mean cell volume (fL), and white blood cell count (10^3^ cells/μL) (Kwon and Belsky 2021). This panel represents a customized variant trained on NHANES data to accommodate available biomarkers in our dataset. Computations strictly followed the published formulas in Kwon and Belsky (2021), including separate training for males and females in KDM, Gompertz proportional hazards modeling in PhenoAge, and appropriate unit conversions (e.g., albumin from g/dL to g/L by multiplying by 0.1; creatinine from mg/dL to μmol/L by multiplying by 88.4 and then log‐transforming). PhenoAge and KDM advancements were calculated as biological ages minus CAs, with positive values indicating accelerated aging and negative values denoting decelerated aging.

### 2.5 Other Covariates

Other covariates were collected including CA, gender, race/ethic, marital state, education level, ratio of family income to poverty and body mass index (BMI). Based on self‐reported questionnaire responses, smoking status (current, former, and never) was determined in accordance with the previously published literature (Song et al. 2025).

Chronic disease status was determined by integrating self‐reported medical history, laboratory test results, or medication use. Diabetes mellitus was identified as a positive answer to the question: “Has a doctor ever told you that you have diabetes?,” HbA1c level > 6.5%, fasting plasma glucose concentration > 7.0 mmol/L, random plasma glucose concentration > 11.1 mmol/L, 2‐h post‐oral glucose tolerance test plasma glucose concentration > 11.1 mmol/L, or self‐reported current use of prescribed diabetes medications (Arslanian et al. 2018). Hypertension was identified if any of the following criteria were met: systolic blood pressure ≥ 140 mmHg in at least 3 measurements, diastolic blood pressure ≥ 90 mmHg in at least 3 measurements, self‐reported use of anti‐hypertensive medications, or a confirmed doctor's diagnosis (Egan 2010). CVD was defined as a positive response to at least one of the following questionnaire items: “Ever told you had heart attack,” “Ever told you had a stroke,” “Ever told had congestive heart failure,” “Ever told you had coronary heart disease,” “Ever told you had angina/angina pectoris” (Chen et al. 2024). Cancer was identified based on a positive response to the questionnaire item “Ever told you had cancer or malignancy.”

### 2.6 Statistical Methods

Weighted descriptive statistics were calculated using the survey R package. Differences between two periodontitis groups (none/mild and moderate/severe) were evaluated by design‐based t‐test for continuous variables, and unweighted Pearson's chi‐square tests for categorical variables.

To estimate survival probabilities across periodontitis groups, we used survey weighted Kaplan–Meier survival analysis (Therneaum and Grambsch 2000; Terry 2026). Survival plots and risk tables were constructed using ggsurvplot (survminer R package), including risk tables displaying the weighted number at risk and the weighted number of events at specified time points (survminer 2025).

Two sets of adjusted models were constructed for all subsequent analyses to comprehensively evaluate the potential confounding effect of chronic systemic diseases. Model 1: Adjusted for CA, gender, race/ethnicity, marital status, education level, ratio of family income to poverty, BMI, and smoking status. Model 2: Further adjusted for diabetes mellitus, hypertension, pre‐existing CVD, and cancer.

To explore the association between periodontitis and biological age advancement, we conducted generalized linear model (GLM) to evaluate the effect of periodontitis on two biological age indicators (stats R package) adjusted by both Model 1 and Model 2. Subgroup analyses were also conducted stratified by age group (< 60 and ≥ 60), gender, and smoking status. Scatter plots were generated to visualize the relationship between CA and biological age across periodontitis groups.

To examine the nonlinear association between biological age advancement (PhenoAge advance and KDM advance) and all‐cause mortality, we used Cox proportional hazards models with restricted cubic splines (RCS) and adjust the model by both Model 1 and Model 2. For each biological age metric, the optimal number of knots (ranging from 3 to 10) was determined via the Akaike Information Criterion, and 3 knots were selected as the best fit for both PhenoAge advance and KDM advance.

Mediation analysis was conducted using the CMAverse R package to evaluate the mediating role of biological age in the association between periodontitis and mortality adjusted by both Model 1 and Model 2 without weighting (Shi et al. 2021). We decomposed the total effect of periodontitis on mortality into three components: total effect, direct effect, and indirect effect. Mediation analyses were performed for all‐cause mortality, CVD‐related mortality, and cancer‐related mortality. Subgroup analyses, stratified age group (< 60 and ≥ 60), gender, and smoking status, were also conducted to explore potential effect modification.

To quantify the robustness of observed associations against unmeasured confounding, *E*‐values were calculated using the EValue R package (VanderWeele and Ding 2017; Mathur and VanderWeele 2020) for both GLM and mediation analysis results with evalues.OLS() and evalues.HR() function respectively.

## 3 Results

### 3.1 Characteristics of the Study Population

After excluding participants without necessary data from 61,594 NHANES participants, this analysis included 16,375 individuals (Figure S1). Table 1 revealed substantial disparities between the no/mild periodontitis group (*n* = 11,448) and moderate/severe periodontitis group (*n* = 4927). The average age of moderate/severe group (55.47 ± 0.34 years) was significantly higher than no/mild group (44.30 ± 0.24 years). There were more female in no/mild group (52.68%) than moderate/severe group (39.54%). Socioeconomic gradients showed lower family income‐to‐poverty ratios and reduced educational attainment in the moderate/severe group. Health profiles revealed significantly higher prevalence of comorbidities among those with moderate/severe periodontitis, including higher rates of diabetes (24.19% vs. 10.72%), hypertension (53.44% vs. 34.55%), and CVD (12.10% vs. 4.96%). Biological age measurements indicated accelerated aging in the moderate/severe group compared to the no/mild group. PhenoAge advance was greater in the moderate/severe group (−0.41 ± 0.10 years) than in the no/mild group (−1.63 ± 0.07 years), and KDM advance was also greater in the moderate/severe group (−0.05 ± 0.15) than in the no/mild group (−0.73 ± 0.10). Mortality outcomes demonstrated a significantly higher death rate (18.31% vs. 10.88%) with shorter follow‐up duration (105.66 ± 1.38 vs. 158.54 ± 2.05 months) in the moderate/severe group.

**Table 1:** The weighted characteristics of participants overall and stratified by periodontitis stage.

		Overall *N* = 16,375	No/Mild *n* = 11,448	Moderate/Severe *n* = 4927	*p*‐value*
**Continuous variables,**	**Age (year)**	46.84 (0.24)	44.30 (0.24)	55.47 (0.34)	< 0.001
**Weighted mean (SD)**	**BMI (kg/m** ^**2**^ **)**	28.55 (0.08)	28.36 (0.09)	29.19 (0.14)	< 0.001
	**Ratio of family income to poverty**	3.14 (0.04)	3.29 (0.04)	2.66 (0.05)	< 0.001
	**PhenoAge advance (year)**	−1.36 (0.06)	−1.63 (0.07)	−0.41 (0.10)	< 0.001
	**KDM advance (year)**	−0.57 (0.09)	−0.73 (0.10)	−0.05 (0.15)	0.013
	**Follow‐up time (month)**	146.54 (1.75)	158.54 (2.05)	105.66 (1.38)	< 0.001
**Categorical variables,**	**Gender**				< 0.001
**Unweighted *n* (%)**	Female	7979 (48.73%)	6031 (52.68%)	1948 (39.54%)	
	Male	8396 (51.27%)	5417 (47.32%)	2979 (60.46%)	
	**Race/ethnicity**				< 0.001
	Mexican American	3024 (18.47%)	2123 (18.54%)	901 (18.29%)	
	Non‐Hispanic Black	3101 (18.94%)	1910 (16.68%)	1191 (24.17%)	
	Non‐Hispanic White	7809 (47.69%)	5884 (51.40%)	1925 (39.07%)	
	Other Hispanic	1186 (7.24%)	758 (6.62%)	428 (8.69%)	
	Other race—including multi‐racial	1255 (7.66%)	773 (6.75%)	482 (9.78%)	
	**Marital status**				< 0.001
	Married	9597 (58.61%)	6844 (59.78%)	2753 (55.88%)	
	No	6778 (41.39%)	4604 (40.22%)	2174 (44.12%)	
	**Education level**				< 0.001
	Above high school	8653 (52.84%)	6617 (57.80%)	2036 (41.32%)	
	high school	5972 (36.47%)	3841 (33.55%)	2131 (43.25%)	
	Less than high school	1750 (10.69%)	990 (8.65%)	760 (15.43%)	
	**Smoking status**				< 0.001
	Former	4077 (24.90%)	2618 (22.87%)	1459 (29.61%)	
	Never	8910 (54.41%)	6749 (58.95%)	2161 (43.86%)	
	Current	3388 (20.69%)	2081 (18.18%)	1307 (26.53%)	
	**Diabetes**				< 0.001
	No	13,956 (85.23%)	10,221 (89.28%)	3735 (75.81%)	
	Yes	2419 (14.77%)	1227 (10.72%)	1192 (24.19%)	
	**Hypertension**				< 0.001
	No	9787 (59.77%)	7493 (65.45%)	2294 (46.56%)	
	Yes	6588 (40.23%)	3955 (34.55%)	2633 (53.44%)	
	**CVD**				< 0.001
	No	15,211 (92.89%)	10,880 (95.04%)	4331 (87.90%)	
	Yes	1164 (7.11%)	568 (4.96%)	596 (12.10%)	
	**Cancer**				< 0.001
	No	15,033 (91.80%)	10,611 (92.69%)	4422 (89.75%)	
	Yes	1342 (8.20%)	837 (7.31%)	505 (10.25%)	
	**Mortality status**				< 0.001
	0	14,227 (86.88%)	10,202 (89.12%)	4025 (81.69%)	
	1	2148 (13.12%)	1246 (10.88%)	902 (18.31%)	
	**Death code**				< 0.001
	Cancer	544 (3.32%)	312 (2.73%)	232 (4.71%)	
	CVD	623 (3.80%)	344 (3.00%)	279 (5.66%)	
	Others	981 (5.99%)	590 (5.15%)	391 (7.94%)	
	NA	14,227 (86.88%)	10,202 (89.12%)	4025 (81.69%)	

* *P*‐values were calculated by survey‐weighted *t*‐test for continuous variables and unweighted Pearson *χ*² test for categorical variables between two periodontitis severity groups.

### 3.2 Path ab+c: Survival Analysis of Periodontitis and Mortality Risks

**Figure 2:**
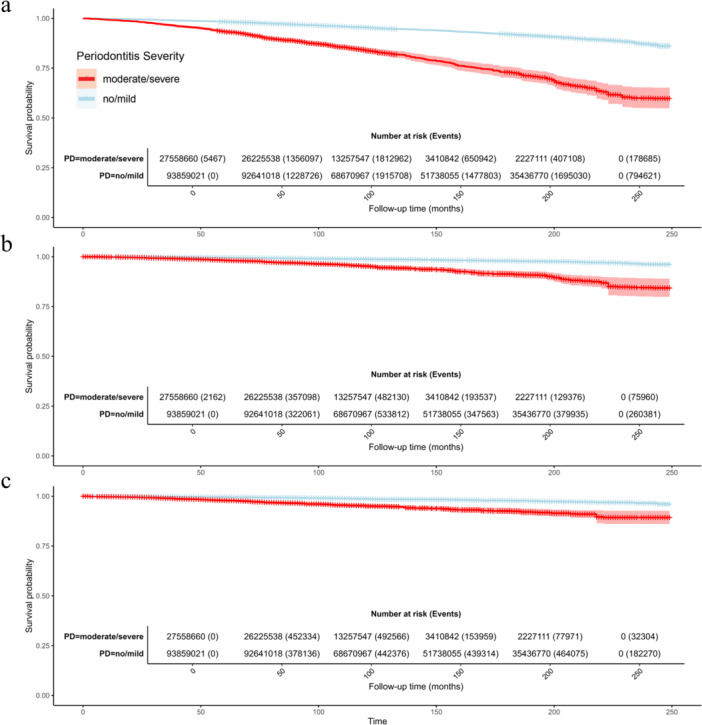
Kaplan–Meier survival curves for mortality by periodontitis severity groups. (a) All‐cause mortality; (b) cardiovascular disease (CVD)‐related mortality; (c) cancer‐related mortality. Curves compare survival probability between the no/mild periodontitis group (blue) and moderate/severe periodontitis group (red) over follow‐up time (months). The weighted number of participants at risk and events for each group at specified time points are presented below each curve.

As shown in Figure 2a, the survival curve of the moderate/severe periodontitis group declined more steeply than that of the no/mild group throughout the 250‐month follow‐up period, indicating a substantially higher all‐cause mortality risk in individuals with moderate/severe periodontitis. This between‐group difference was statistically significant (log‐rank test, *p* < 0.001). According to the weighted risk table, there were 93,859,021 individuals at risk in the no/mild group and 27,558,660 in the moderate/severe group at baseline. By 150 months, the weighted number at risk had dropped to 51,738,055 and 3,410,842 respectively. A similar trend was observed for CVD‐related and cancer‐related mortality (Figure 2b,c), where the survival curve of the moderate/severe periodontitis group showed a more pronounced downward slope compared to the no/mild group, with the between‐group disparity also achieving statistical significance (log‐rank test, *p* < 0.001).

### 3.3 Path a: Association Between Periodontitis and Biological Age Advances

**Figure 3:**
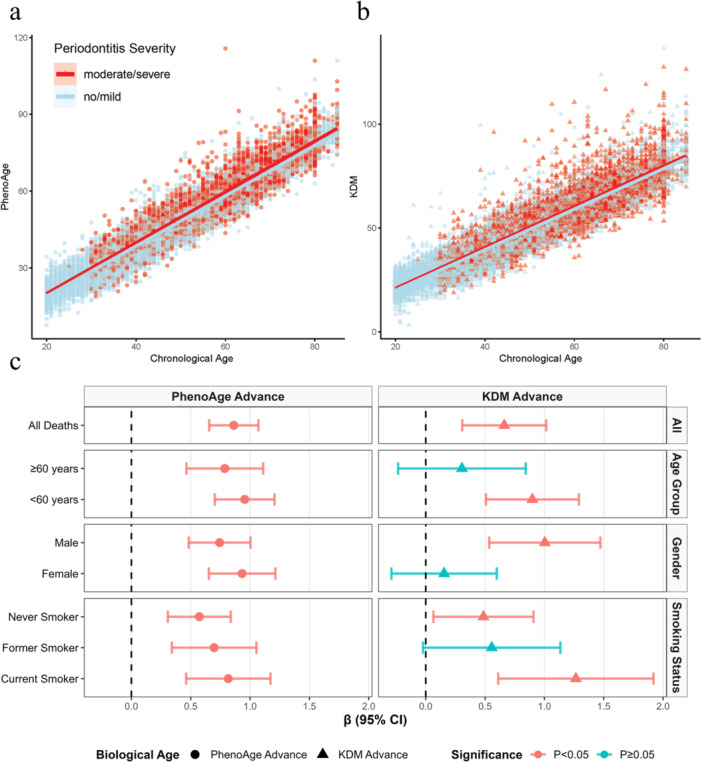
Association between periodontitis severity, chronological age, and biological age. (a, b) Scatter plots with linear regression lines depict the relationship between chronological age and PhenoAge and KDM, stratified by periodontitis severity group. (c) Forest plot of β coefficients from generalized linear models assessing the association between periodontitis and biological age advancement across overall population and subgroups adjusted by chronological age, gender, race/ethic, marital status, education level, ratio of family income to poverty, and BMI. Circular markers represent PhenoAge advance; triangular markers represent KDM advance. Magenta bars indicate statistical significance (*p* < 0.05, 95% CIs do not cross the null *β* = 1); indigo bars indicate non‐significance (*p* ≥ 0.05, 95% CIs cross *β* = 1).

To investigate the association between periodontitis and biological age advancement, we analyzed two biological age indicators using scatter plots and GLM. Scatter plots (Figure 3a,b) showed that the regression lines for the moderate/severe periodontitis group lay above those for the no/mild periodontitis group, indicating greater biological age advancement in individuals with moderate/severe periodontitis at any given CA. GLM results adjusted by Model 1 (Figure 3c and Table S2) demonstrated that moderate/severe periodontitis group was linked to a 0.863‐unit increase in PhenoAge advance (95% CI: 0.655–1.070, *p* < 0.001) and a 0.661‐unit increase in KDM advance (95% CI: 0.307–1.015, *p* < 0.001) in the overall population. Subgroup analyses proved that the association of moderate/severe periodontitis with PhenoAge advance was significant across age, gender, and smoking status strata. For KDM advance, significant associations were observed in younger adults ( < 60 years), males, never and current smokers, while some subgroups (e.g., ≥ 60 years, females, former smokers) showed non‐significant trends. GLM results adjusted by Model 2 showed similar findings, except that the never smokers’ group was non‐significant (Figure S2).

### 3.4 Path b: Association Between Biological Age and Mortality

**Figure 4:**
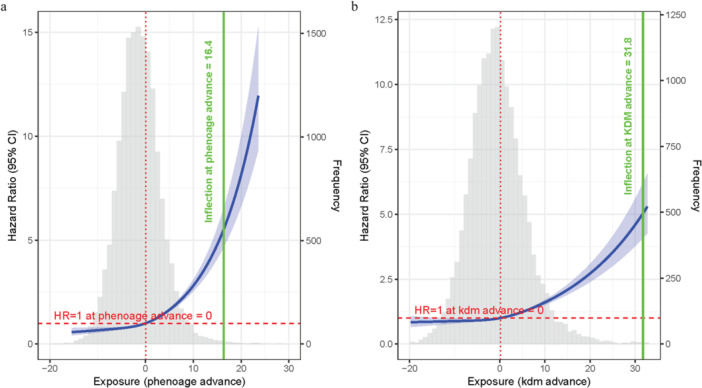
Restricted cubic spline curves from Cox proportional hazards models illustrating the nonlinear association between biological age advancement and all‐cause mortality. (a) Association for PhenoAge advance; (b) association for KDM advance adjusted by chronological age, gender, race/ethic, marital status, education level, ratio of family income to poverty, and BMI. Blue curves represent hazard ratios (HRs) with 95% confidence intervals. Gray histograms depict the frequency distribution of biological age advancement. Dashed red lines indicate the reference HR = 1; dotted red lines mark the points where HR = 1; solid green lines and labels denote approximate inflection points where risk increases more steeply.

Cox proportional hazards models with RCS revealed a significant, nonlinear association between biological age advancement and all‐cause mortality, consistent across both PhenoAge and KDM metrics (overall *p* < 0.001, nonlinear *p* < 0.001 for both) in Model 1 (Figure 4) and Model 2 (Figure S3). The overall pattern was J‐shaped: mortality risk remained relatively stable when biological age was at or below the population average (HR = 1 reference point), while increased sharply with further advancement. This sharp increase was more pronounced for PhenoAge advance, with an inflection point at approximately 16.4 years, compared to 31.8 years for KDM advance (Figure 4). Consistent with this pattern, the distribution of biological age advancement (gray histograms) showed that most participants clustered in the lower‐risk range, while the upper tail of the distribution was associated with a substantially elevated mortality hazard.

### 3.5 Path ab: Mediation Role of Biological Age Advances in the Association Between Periodontitis and Mortality

**Figure 5:**
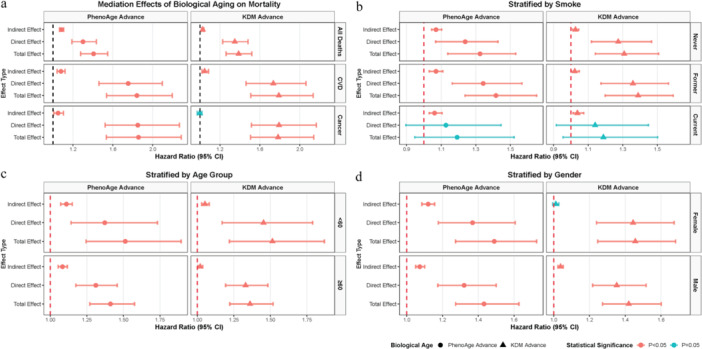
Forest plots display hazard ratios (HRs) with 95% confidence intervals from mediation analyses. For each biological age metric (PhenoAge advance [left panels]; KDM advance [right panels]), bars are ordered from top to bottom as: indirect effect, direct effect, and total effect. Stratification: Analyses are stratified by: (a) mortality outcome (all‐cause, CVD‐related, cancer‐related) in the total population; (b) smoking status (never, former, current) for all‐cause mortality; (c) age group (< 60 years, ≥ 60 years) for all‐cause mortality; (d) gender (female, male) for all‐cause mortality. All models are adjusted for chronological age, gender, race/ethnicity, marital status, education level, ratio of family income to poverty, and BMI (Model 1). Circular markers represent PhenoAge advance; triangular markers represent KDM advance. Magenta bars indicate statistical significance (*p* < 0.05, 95% CIs do not cross the null HR = 1); indigo bars indicate non‐significance (*p* ≥ 0.05, 95% CIs cross HR = 1).

Mediation analysis adjusted by Model 1 (Figure 5 and Table S3) revealed that biological age, especially PhenoAge consistently mediated the association between periodontitis severity and mortality. As shown in Figure 5a, significant total, direct, and indirect effects of periodontitis on all‐cause and CVD‐related mortality were observed via PhenoAge advance and KDM advance. In cancer‐related mortality, PhenoAge advance exhibited significant direct and indirect effects, while KDM advance had a non‐significant indirect effect. The proportion mediated by PhenoAge advance was approximately 27% (95% CI: 21%–36%) for all‐cause mortality and 16% (95% CI: 9%–24%) for CVD‐related mortality, whereas for KDM advance, it was 9% (95% CI: 5%–15%) for all‐cause mortality and 10% (95% CI: 4%–17%) for CVD‐related mortality (Table S3), indicating that biological age explains a modest portion of the periodontitis‐mortality association overall. Subgroup analyses (Figure 5b–d) revealed that PhenoAge and KDM advances mediated effects across most age, gender, and smoking strata except in KDM advances in female participants. Intriguingly, among current smokers, despite non‐significant total and direct effects, the indirect effect remained significant for both biological age metrics. When covariates were further adjusted for systemic diseases (Model 2, Figure S4), the mediating role of PhenoAge in the periodontitis‐mortality association remained robust and largely consistent with Model 1. In contrast, the mediating effects of KDM advance showed reduced significance in CVD‐related mortality and several subgroups (e.g., female participants, those aged ≥ 60 years) compared to Model 1.

## 4 Discussion

This study analyzed data from 16,375 participants across six cycles of NHANES to support our conceptual mediation model, in which periodontitis is associated with mortality risk directly and indirectly through accelerated biological aging (Figure 1).

We systematically examined the model's pathways. First, moderate/severe periodontitis was associated with significantly higher all‐cause, CVD, and cancer mortality (Path ab+c, Figure 2). These findings align with previous epidemiological evidence linking periodontitis to increased mortality (Larvin et al. 2024; Romandini et al. 2020). Second, periodontitis was linked to greater advancement in both PhenoAge and KDM biological age metrics (Path a, Figure 3 and Figure S2), with PhenoAge showing more consistent associations across subgroups. This conclusion is consistent with recent research indicating that periodontitis is tied to accelerated biological aging (Li, Wen et al. 2024; Huang et al. 2024). Third, biological age advancement itself exhibited a strong, nonlinear relationship with mortality risk, increasing sharply beyond thresholds (Path b, Figure 4 and Figure S3). Finally, mediation analyses indicated that biological age, particularly PhenoAge, partially mediated the periodontitis‐mortality association, accounting for approximately 27% for PhenoAge and 9% for KDM of the total effect for all‐cause mortality (Path ab, Figure 5 and Figure S4). This partial mediation was observed across most subgroups, though the proportion mediated varied.

The indirect effect via biological aging, while statistically significant, was modest in magnitude. This can be explained by a key nonlinearity: periodontitis was associated with a mean biological age increase of < 1 year, whereas mortality risk rose sharply after advancement exceeded approximately 16 years for PhenoAge and approximately 32 years for KDM. This mismatch dilutes the measurable indirect effect. Furthermore, residual risk likely operates through other pathways, including inflammatory, metabolic, or socioeconomic factors. The attenuation of indirect effects in models adjusted for systemic comorbidities (Model 2) is likely due to collider bias, as conditions like diabetes and CVD may act as sequential mediators rather than mere confounders, blocking part of the natural pathway when conditioned upon (Tönnies et al. 2022).

Two methodological factors may influence our estimates. First, the shift from partial‐ to FMPEs across NHANES cycles, combined with binary disease classification, may introduce non‐differential misclassification bias, potentially attenuating associations (Eke et al. 2010; Duong et al. 2016). Second, the observed disparity between PhenoAge and KDM may stem from their methodological differences and sensitivity to NHANES’ age topcoding. PhenoAge, which maps biomarker profiles to mortality risk, is less vulnerable to topcoding‐induced compression of the age distribution than KDM, which relies on linear regression against CA.

Our study has several limitations. Its observational design precludes causal inference, and we cannot rule out reverse causation or unmeasured confounding by shared genetic or lifestyle factors. Due to software constraints, mediation analyses could not incorporate NHANES survey weights, which may limit the generalizability of those estimates to the US population and potentially attenuate associations. Finally, the use of a complete‐case analysis, while necessary for biological age computation, may introduce bias if data were not missing completely at random.

Despite these limitations, our findings carry clinical relevance. Integrating oral health screening with biological age assessment (using routine biomarkers) into the care of high‐risk groups, such as older adults with multimorbidity or long‐term smokers, could help identify individuals who may benefit most from targeted periodontal interventions and monitoring of aging trajectories (Sanz et al. 2020; Sumayin Ngamdu et al. 2022). Future research could employ emerging biological age tools (e.g., microbiome [Galkin et al. 2020] or glycomic clocks [Machin et al. 2018]) to explore mechanistic links, utilize prospective and interventional designs to establish causality, and test whether periodontal therapy can decelerate biological aging.

## Author Contributions

Dawei Zhang contributed to the conception, methodology, formal analysis, visualization, and writing the original draft. Shijie Zhu contributed to methodology, formal analysis, software, and visualization. Patrick Rijkschroeff contributed to conception, supervision, and project administration. George Pelekos and Lijian Jin contributed to review, supervision, and gave the final approval of the version to be published. All authors have made substantial contributions to the conception and design of the study and gave their final approval and agree to be accountable for all aspects of the work.

## Funding

The authors received no specific funding for this work.

## Conflicts of Interest

The authors declare no conflicts of interest.

## Supporting information


**Table S1:** STROBE Statement—checklist of items that should be included in reports of observational studies. **Table S2:** Generalized Linear Model Results for Periodontitis Severity and Biological Age Advancement Association and E‐values in Total Population and Stratified Subgroups. **Table S3:** Mediation Analysis of Biological Age in The Association Between Periodontitis Severity and Mortality and E‐values in Total Population and Stratified Subgroups.

## Data Availability

The data that support the findings of this study are openly available in National Health and Nutrition Examination Survey at https://wwwn.cdc.gov/nchs/nhanes/default.aspx.
