# Supplementary material for: The Mediating Role of Biological Age Advance in the Association Between Periodontitis and Mortality: Biological Aging Links Periodontitis to Mortality
Source: Clin Exp Dent Res. 2026 Feb 9;12(1):e70305. doi: 10.1002/cre2.70305 (PMC12887437; doi:10.1002/cre2.70305)
Supplement: Supplementary file 1 — Table S1: STROBE Statement—checklist of items that should be included in reports of observational studies. Table S2: Generalized Linear Model Results for Periodontitis Severity and Biological Age Advancement Association and E‐values in Total Population and Stratified Subgroups. Table S3: Mediation Analysis of Biological Age in The Association Between Periodontitis Severity and Mortality and E‐values in Total Population and Stratified Subgroups. [file CRE2-12-e70305-s001.docx]

**The Mediating Role of Biological Age Advance in the Association Between Periodontitis and Mortality​​**

Dawei Zhang^1^, Shijie Zhu^2,3^, George Pelekos^1^, Lijian Jin^1^, Patrick Rijkschroeff^1^

1. Division of Periodontology and Implant Dentistry, Faculty of Dentistry, The University of Hong Kong, No. 34, Hospital Road, Sai Ying Pun, Hong Kong, China
2. School of Public Health, LKS Faculty of Medicine, The University of Hong Kong, Pok Fu Lam, Hong Kong, China
3. Quality Management and Accreditation Department, The University of Hong Kong-Shenzhen Hospital, Shenzhen 518053, China

Table S1. STROBE Statement—checklist of items that should be included in reports of observational studies

|  | Item No | Recommendation | Page  No |
| --- | --- | --- | --- |
| **Title and abstract** | 1 | (*a*) Indicate the study’s design with a commonly used term in the title or the abstract | 1 |
|  |  | (*b*) Provide in the abstract an informative and balanced summary of what was done and what was found | 2 |
| Introduction | | | |
| Background/rationale | 2 | Explain the scientific background and rationale for the investigation being reported | 3 |
| Objectives | 3 | State specific objectives, including any prespecified hypotheses | 4 |
| Methods | | | |
| Study design | 4 | Present key elements of study design early in the paper | 5 |
| Setting | 5 | Describe the setting, locations, and relevant dates, including periods of recruitment, exposure, follow-up, and data collection | 5-6 |
| Participants | 6 | (*a*) *Cohort study*—Give the eligibility criteria, and the sources and methods of selection of participants. Describe methods of follow-up  *Case-control study*—Give the eligibility criteria, and the sources and methods of case ascertainment and control selection. Give the rationale for the choice of cases and controls  *Cross-sectional study*—Give the eligibility criteria, and the sources and methods of selection of participants | 5-7 |
|  |  | (*b*) *Cohort study*—For matched studies, give matching criteria and number of exposed and unexposed  *Case-control study*—For matched studies, give matching criteria and the number of controls per case |  |
| Variables | 7 | Clearly define all outcomes, exposures, predictors, potential confounders, and effect modifiers. Give diagnostic criteria, if applicable | 5-7 |
| Data sources/ measurement | 8 | For each variable of interest, give sources of data and details of methods of assessment (measurement). Describe comparability of assessment methods if there is more than one group | 5-7 |
| Bias | 9 | Describe any efforts to address potential sources of bias | 5-7 |
| Study size | 10 | Explain how the study size was arrived at | Figure S1 |
| Quantitative variables | 11 | Explain how quantitative variables were handled in the analyses. If applicable, describe which groupings were chosen and why | 6-7 |
| Statistical methods | 12 | (*a*) Describe all statistical methods, including those used to control for confounding | 6-7 |
|  |  | (*b*) Describe any methods used to examine subgroups and interactions | 7 |
|  |  | (*c*) Explain how missing data were addressed | Figure S1 |
|  |  | (*d*) *Cohort study*—If applicable, explain how loss to follow-up was addressed  *Case-control study*—If applicable, explain how matching of cases and controls was addressed  *Cross-sectional study*—If applicable, describe analytical methods taking account of sampling strategy | 5 |
|  |  | (*e*) Describe any sensitivity analyses |  |

| Results | | | |
| --- | --- | --- | --- |
| Participants | 13 | (a) Report numbers of individuals at each stage of study—eg numbers potentially eligible, examined for eligibility, confirmed eligible, included in the study, completing follow-up, and analysed | Table 1 |
|  |  | (b) Give reasons for non-participation at each stage |  |
|  |  | (c) Consider use of a flow diagram | Figure S1 |
| Descriptive data | 14 | (a) Give characteristics of study participants (eg demographic, clinical, social) and information on exposures and potential confounders | Table 1 |
|  |  | (b) Indicate number of participants with missing data for each variable of interest | Figure S1 |
|  |  | (c) *Cohort study*—Summarise follow-up time (eg, average and total amount) | Table 1 |
| Outcome data | 15 | *Cohort study*—Report numbers of outcome events or summary measures over time | Table 1 |
|  |  | *Case-control study—*Report numbers in each exposure category, or summary measures of exposure |  |
|  |  | *Cross-sectional study—*Report numbers of outcome events or summary measures |  |
| Main results | 16 | (*a*) Give unadjusted estimates and, if applicable, confounder-adjusted estimates and their precision (eg, 95% confidence interval). Make clear which confounders were adjusted for and why they were included | Figure 3 and 5 |
|  |  | (*b*) Report category boundaries when continuous variables were categorized |  |
|  |  | (*c*) If relevant, consider translating estimates of relative risk into absolute risk for a meaningful time period | Figure 2 |
| Other analyses | 17 | Report other analyses done—eg analyses of subgroups and interactions, and sensitivity analyses | Figure 3 and 5 |
| Discussion | | | |
| Key results | 18 | Summarise key results with reference to study objectives | 16 |
| Limitations | 19 | Discuss limitations of the study, taking into account sources of potential bias or imprecision. Discuss both direction and magnitude of any potential bias | 17-18 |
| Interpretation | 20 | Give a cautious overall interpretation of results considering objectives, limitations, multiplicity of analyses, results from similar studies, and other relevant evidence | 17-18 |
| Generalisability | 21 | Discuss the generalisability (external validity) of the study results | 18 |
| Other information | | | |
| Funding | 22 | Give the source of funding and the role of the funders for the present study and, if applicable, for the original study on which the present article is based | Title Page |

Table S2 Generalized Linear Model Results for Periodontitis Severity and Biological Age Advancement Association and E-values in Total Population and Stratified Subgroups^†^

| Biological Age | Subgroup | Effect Size | 95% CI | E-value | 95% CI |
| --- | --- | --- | --- | --- | --- |
| KDM Advance | Total People | 0.661 | 0.307–1.015 | 1.427 | 1.264–NA |
| PhenoAge Advance | Total People | 0.863 | 0.655–1.07 | 1.705 | 1.576–NA |
| KDM Advance | <60 Years | 0.899 | 0.507–1.291 | 1.579 | 1.392–NA |
| PhenoAge Advance | <60 Years | 0.955 | 0.703–1.207 | 1.804 | 1.636–NA |
| KDM Advance | ≥60 Years | 0.305 | -0.233–0.843 | 1.229 | 1–NA |
| PhenoAge Advance | ≥60 Years | 0.787 | 0.464–1.11 | 1.605 | 1.42–NA |
| KDM Advance | Male | 1.003 | 0.535–1.472 | 1.562 | 1.368–NA |
| PhenoAge Advance | Male | 0.743 | 0.483–1.003 | 1.637 | 1.47–NA |
| KDM Advance | Female | 0.154 | -0.29–0.599 | 1.176 | 1–NA |
| PhenoAge Advance | Female | 0.933 | 0.653–1.214 | 1.743 | 1.57–NA |
| KDM Advance | Never Smoker | 0.486 | 0.064–0.908 | 1.352 | 1.113–NA |
| PhenoAge Advance | Never Smoker | 0.572 | 0.307–0.837 | 1.526 | 1.348–NA |
| KDM Advance | Former Smoker | 0.556 | -0.023–1.135 | 1.361 | 1–NA |
| PhenoAge Advance | Former Smoker | 0.697 | 0.341–1.054 | 1.593 | 1.365–NA |
| KDM Advance | Current Smoker | 1.265 | 0.611–1.919 | 1.721 | 1.43–NA |
| PhenoAge Advance | Current Smoker | 0.817 | 0.461–1.172 | 1.682 | 1.455–NA |

^†^ Adjusted by chronological age, gender, race/ethic, marital status, education level, ratio of family income to poverty, and BMI.

Table S3 Mediation Analysis of Biological Age in The Association Between Periodontitis Severity and Mortality and E-values in Total Population and Stratified Subgroups^†^

| Biological Age | Outcome | Subgroup | Effect Type | HR | HR 95% CI | E-value | E-value 95% CI |
| --- | --- | --- | --- | --- | --- | --- | --- |
| PhenoAge | All-cause Mortality | Total People | Direct Effect | 1.300 | 1.181–1.424 | 1.924 | 1.924–NA |
|  |  |  | Indirect Effect | 1.085 | 1.067–1.106 | 1.388 | 1.388–NA |
|  |  |  | Total Effect | 1.406 | 1.279–1.536 | 2.162 | 2.162–NA |
|  |  |  | Proportion Mediated | 0.270 | 0.207–0.358 |  |  |
| KDM | All-cause Mortality | Total People | Direct Effect | 1.349 | 1.23–1.48 | 2.034 | 2.034–NA |
|  |  |  | Indirect Effect | 1.027 | 1.016–1.04 | 1.193 | 1.193–NA |
|  |  |  | Total Effect | 1.387 | 1.272–1.525 | 2.120 | 2.12–NA |
|  |  |  | Proportion Mediated | 0.094 | 0.054–0.148 |  |  |
| PhenoAge | CVD-related Mortality | Total People | Direct Effect | 1.753 | 1.461–2.085 | 2.901 | 2.901–NA |
|  |  |  | Indirect Effect | 1.080 | 1.040–1.130 | 1.373 | 1.373–NA |
|  |  |  | Total Effect | 1.840 | 1.554–2.179 | 3.083 | 3.083–NA |
|  |  |  | Proportion Mediated | 0.161 | 0.090–0.243 |  |  |
| KDM | CVD-related Mortality | Total People | Direct Effect | 1.733 | 1.470–2.050 | 2.859 | 2.859–NA |
|  |  |  | Indirect Effect | 1.047 | 1.018–1.086 | 1.268 | 1.268–NA |
|  |  |  | Total Effect | 1.788 | 1.523–2.122 | 2.975 | 2.975–NA |
|  |  |  | Proportion Mediated | 0.101 | 0.044–0.170 |  |  |
| PhenoAge | Cancer-related Mortality | Total People | Direct Effect | 1.852 | 1.547–2.221 | 3.109 | 3.109–NA |
|  |  |  | Indirect Effect | 1.051 | 1.006–1.096 | 1.281 | 1.281–NA |
|  |  |  | Total Effect | 1.859 | 1.542–2.206 | 3.123 | 3.123–NA |
|  |  |  | Proportion Mediated | 0.104 | 0.017–0.210 |  |  |
| KDM | Cancer-related Mortality | Total People | Direct Effect | 1.793 | 1.521–2.181 | 2.985 | 2.985–NA |
|  |  |  | Indirect Effect | 0.998 | 0.972–1.019 | 1.045 | 1.045–NA |
|  |  |  | Total Effect | 1.783 | 1.507–2.158 | 2.964 | 2.964–NA |
|  |  |  | Proportion Mediated | -0.004 | -0.067–0.044 |  |  |
| PhenoAge | All-cause Mortality | Current Smoker | Direct Effect | 1.127 | 0.897–1.456 | 1.506 | 1.506–NA |
|  |  |  | Indirect Effect | 1.062 | 1.03–1.106 | 1.317 | 1.317–NA |
|  |  |  | Total Effect | 1.192 | 0.939–1.527 | 1.671 | 1.671–NA |
|  |  |  | Proportion Mediated | 0.360 | -2.926–2.814 |  |  |
| KDM | All-cause Mortality | Current Smoker | Direct Effect | 1.140 | 0.879–1.456 | 1.540 | 1.54–NA |
|  |  |  | Indirect Effect | 1.038 | 1.012–1.069 | 1.235 | 1.235–NA |
|  |  |  | Total Effect | 1.188 | 0.923–1.514 | 1.661 | 1.661–NA |
|  |  |  | Proportion Mediated | 0.230 | -0.765–2.678 |  |  |
| PhenoAge | All-cause Mortality | Former Smoker | Direct Effect | 1.344 | 1.155–1.571 | 2.025 | 2.025–NA |
|  |  |  | Indirect Effect | 1.070 | 1.033–1.112 | 1.343 | 1.343–NA |
|  |  |  | Total Effect | 1.418 | 1.218–1.651 | 2.189 | 2.189–NA |
|  |  |  | Proportion Mediated | 0.222 | 0.102–0.381 |  |  |
| KDM | All-cause Mortality | Former Smoker | Direct Effect | 1.359 | 1.161–1.562 | 2.057 | 2.057–NA |
|  |  |  | Indirect Effect | 1.023 | 1.003–1.046 | 1.174 | 1.174–NA |
|  |  |  | Total Effect | 1.389 | 1.183–1.595 | 2.123 | 2.123–NA |
|  |  |  | Proportion Mediated | 0.079 | 0.009–0.187 |  |  |
| PhenoAge | All-cause Mortality | Never Smoker | Direct Effect | 1.239 | 1.072–1.456 | 1.782 | 1.782–NA |
|  |  |  | Indirect Effect | 1.070 | 1.045–1.101 | 1.343 | 1.343–NA |
|  |  |  | Total Effect | 1.324 | 1.147–1.558 | 1.980 | 1.98–NA |
|  |  |  | Proportion Mediated | 0.267 | 0.161–0.526 |  |  |
| KDM | All-cause Mortality | Never Smoker | Direct Effect | 1.273 | 1.105–1.449 | 1.864 | 1.864–NA |
|  |  |  | Indirect Effect | 1.027 | 1.011–1.046 | 1.193 | 1.193–NA |
|  |  |  | Total Effect | 1.308 | 1.143–1.491 | 1.943 | 1.943–NA |
|  |  |  | Proportion Mediated | 0.111 | 0.044–0.226 |  |  |
| PhenoAge | All-cause Mortality | <60 Years | Direct Effect | 1.373 | 1.101–1.722 | 2.088 | 2.088–NA |
|  |  |  | Indirect Effect | 1.110 | 1.075–1.150 | 1.459 | 1.459–NA |
|  |  |  | Total Effect | 1.513 | 1.215–1.900 | 2.394 | 2.394–NA |
|  |  |  | Proportion Mediated | 0.292 | 0.174–0.535 |  |  |
| KDM | All-cause Mortality | <60 Years | Direct Effect | 1.455 | 1.171–1.801 | 2.268 | 2.268–NA |
|  |  |  | Indirect Effect | 1.053 | 1.030–1.081 | 1.288 | 1.288–NA |
|  |  |  | Total Effect | 1.515 | 1.221–1.874 | 2.399 | 2.399–NA |
|  |  |  | Proportion Mediated | 0.147 | 0.078–0.305 |  |  |
| PhenoAge | All-cause Mortality | ≥60 Years | Direct Effect | 1.310 | 1.166–1.461 | 1.948 | 1.948–NA |
|  |  |  | Indirect Effect | 1.083 | 1.053–1.117 | 1.384 | 1.384–NA |
|  |  |  | Total Effect | 1.411 | 1.260–1.578 | 2.172 | 2.172–NA |
|  |  |  | Proportion Mediated | 0.264 | 0.181–0.382 |  |  |

^†^ Adjusted by chronological age, gender, race/ethic, marital status, education level, ratio of family income to poverty, and BMI.


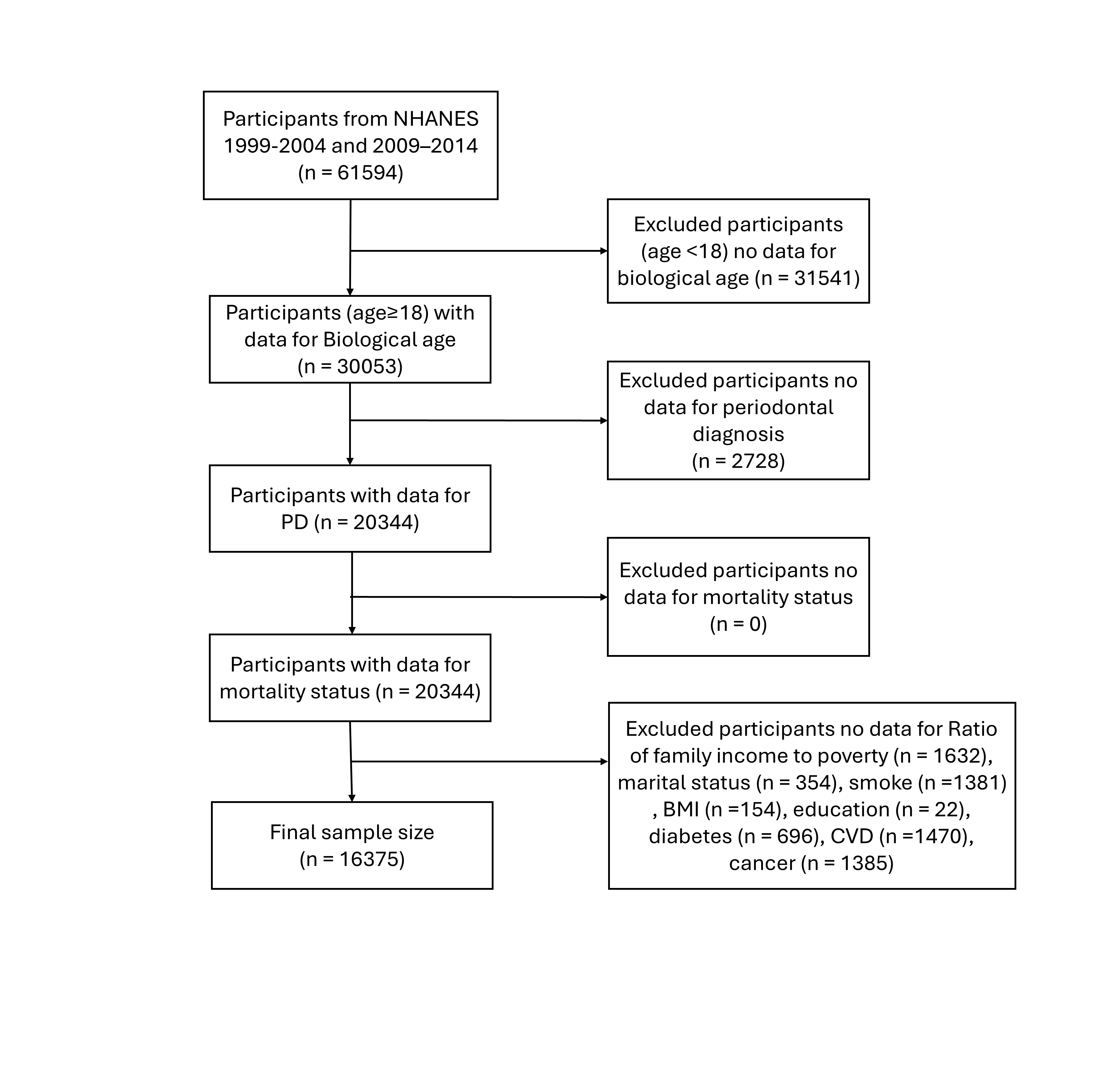


Figure S1. Flow chart indicating the subset of participants included in the analysis


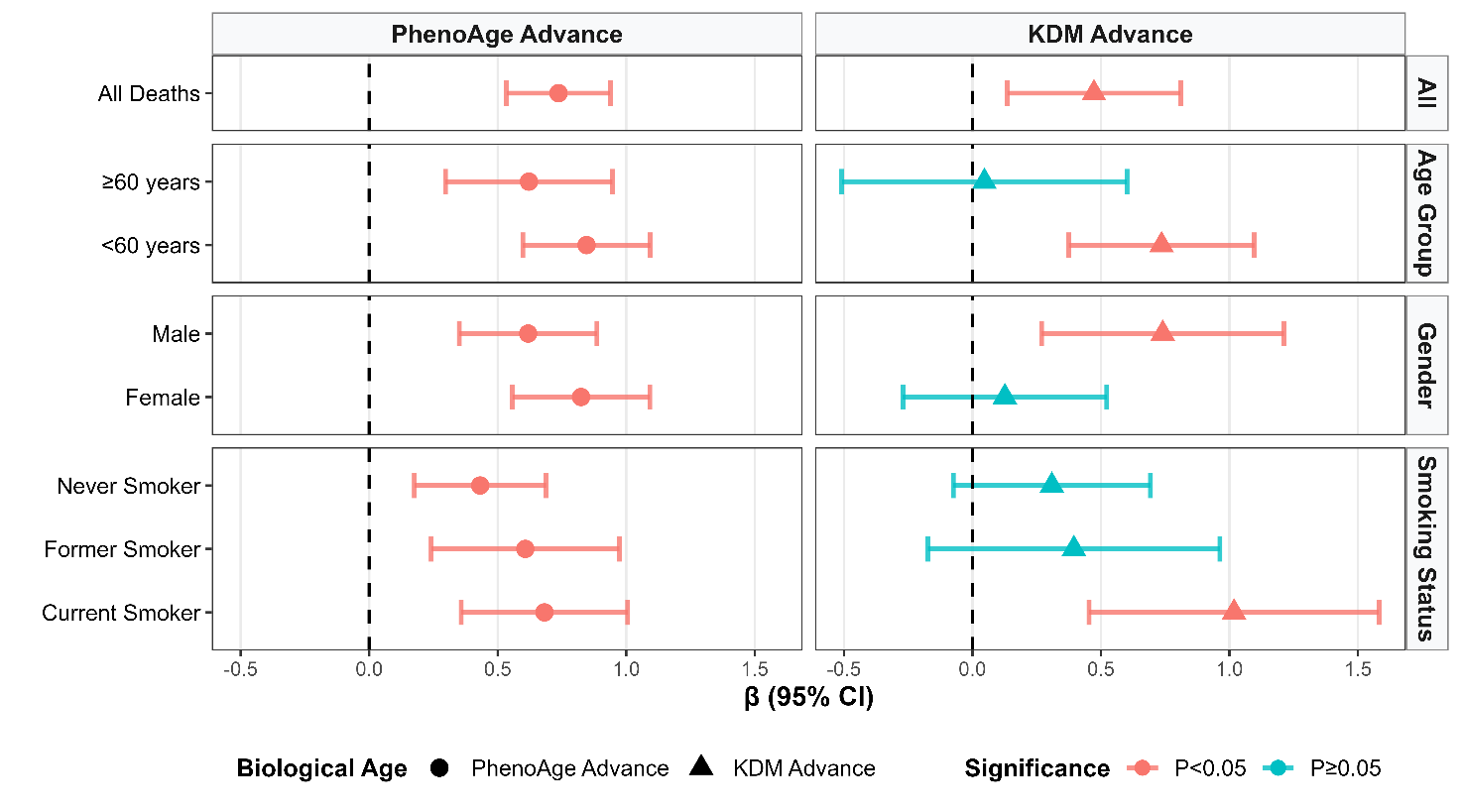


Figure S2. Forest plot of β coefficients from generalized linear models assessing the association between periodontitis severity and biological age advancement across overall population and subgroups adjusted by chronological age, gender, race/ethic, marital status, education level, ratio of family income to poverty, BMI, diabetes, cardiovascular disease, hypertension and cancer. Circular markers represent PhenoAge Advance; triangular markers represent KDM Advance. Magenta bars indicate statistical significance (P<0.05, 95% CIs do not cross the null β=1); indigo bars indicate non-significance (P≥0.05, 95% CIs cross β=1).


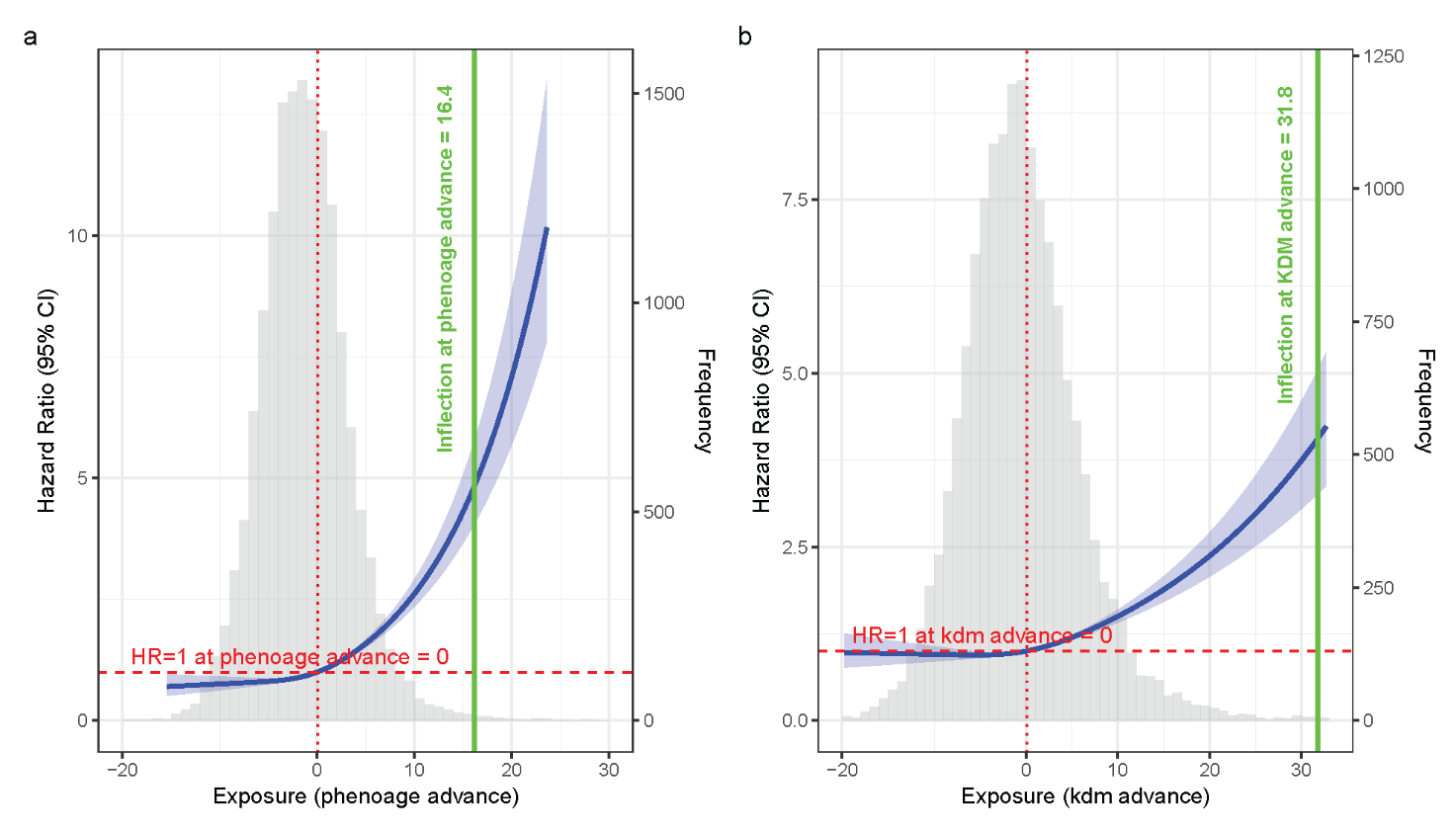


Figure S3. Restricted cubic spline curves from Cox proportional hazards models illustrating the nonlinear association between biological age advancement and all - cause mortality. (a) Association for PhenoAge Advance; (b) Association for KDM Advance adjusted by chronological age, gender, race/ethic, marital status, education level, ratio of family income to poverty, BMI, diabetes, cardiovascular disease, hypertension and cancer. Blue curves represent hazard ratios (HRs) with 95% confidence intervals. Grey histograms depict the frequency distribution of biological age advancement. Dashed red lines indicate the reference HR=1; dotted red lines mark the points where HR=1; solid green lines and labels denote approximate inflection points where risk increases more steeply.


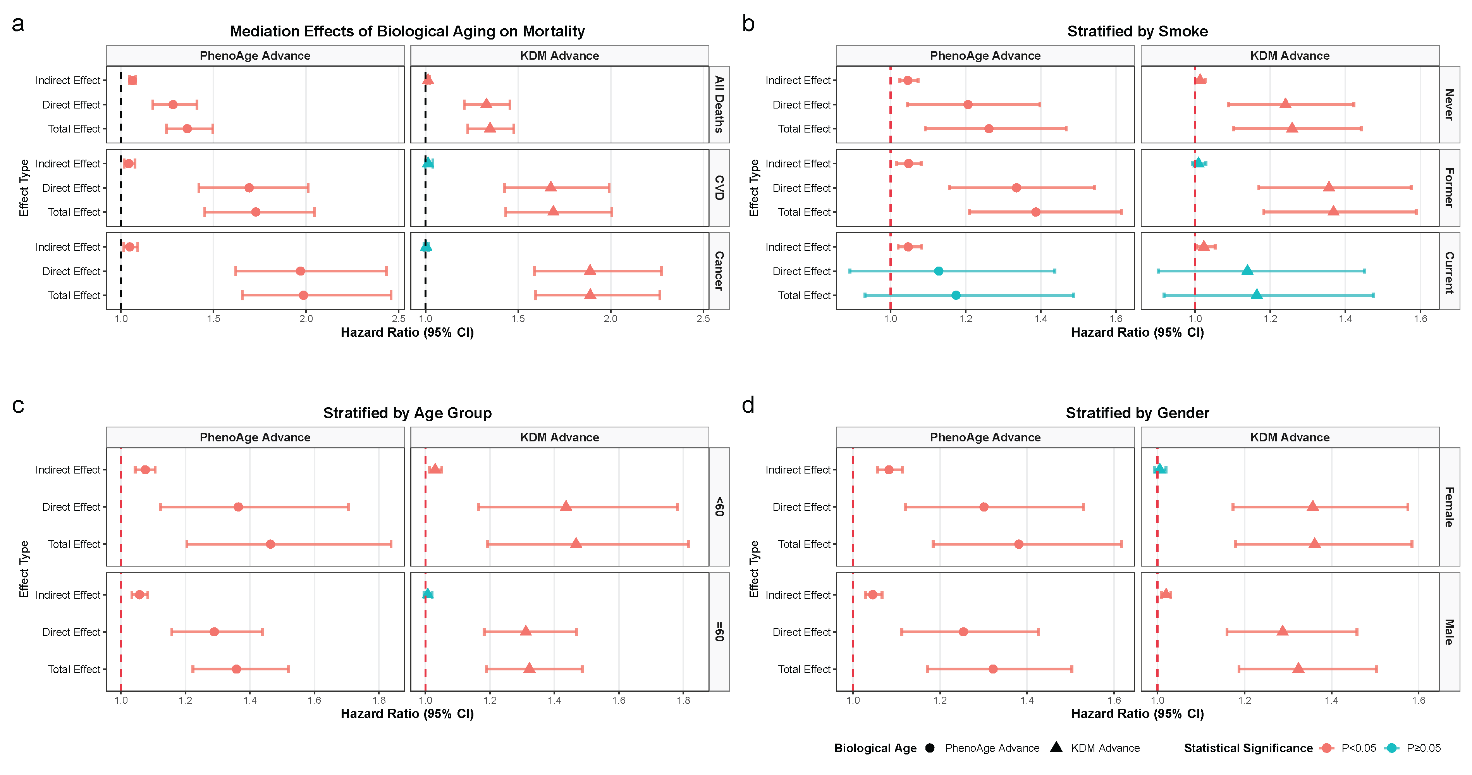


Figure S4. Forest plots display hazard ratios (HRs) with 95% confidence intervals from mediation analyses. For each biological age metric [PhenoAge Advance (left panels); KDM Advance (right panels)], bars are ordered from top to bottom as: indirect effect, direct effect, and total effect. Stratification: Analyses are stratified by: (a) mortality outcome (all-cause, CVD-related, cancer-related) in the total population; (b) smoking status (never, former, current) for all-cause mortality; (c) age group (<60 years, ≥60 years) for all-cause mortality; (d) gender (female, male) for all-cause mortality. All models are adjusted for chronological age, gender, race/ethnicity, marital status, education level, ratio of family income to poverty, BMI, diabetes, cardiovascular disease, hypertension and cancer (Model 2). Circular markers represent PhenoAge Advance; triangular markers represent KDM Advance. Magenta bars indicate statistical significance (P<0.05, 95% CIs do not cross the null HR=1); indigo bars indicate non-significance (P≥0.05, 95% CIs cross HR=1).


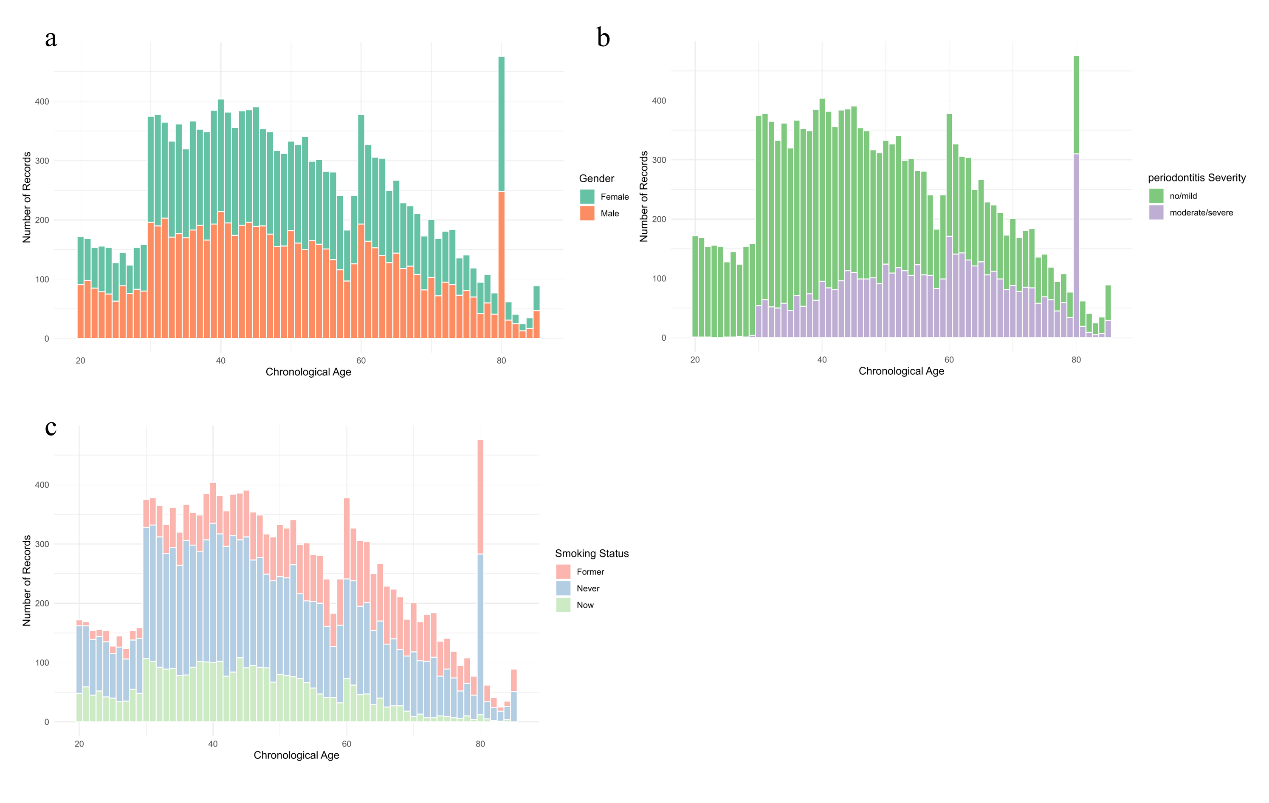


Figure S5. Distribution of chronological age across subgroups in the study population. (a) Histogram of age stratified by gender. (b) Histogram of age stratified by periodontitis severity. (c) Histogram of age stratified by smoking status.
